# Shear and Extensional Rheology of Linear and Branched Polybutylene Succinate Blends

**DOI:** 10.3390/polym13040652

**Published:** 2021-02-22

**Authors:** Violette Bourg, Rudy Valette, Nicolas Le Moigne, Patrick Ienny, Valérie Guillard, Anne Bergeret

**Affiliations:** 1 Polymers Composites and Hybrids (PCH)-IMT Mines Ales, 30100 Ales, France; nicolas.le-moigne@mines-ales.fr (N.L.M.); anne.bergeret@mines-ales.fr (A.B.); 2 LMGC, IMT Mines Ales, University of Montpellier, CNRS, 30100 Ales, France; patrick.ienny@mines-ales.fr; 3 Ingénierie des Agropolymères et Technologie Emergentes—IATE, Univ Montpellier, INRAE, Institut Agro, 34000 Montpellier, France; valerie.guillard@umontpellier.fr; 4 Nestlé Research SA, Nestlé Institute of Packaging Sciences, Route du Jorat, 57, 1000 Lausanne, Switzerland; 5 MINES ParisTech, CEMEF-Centre de Mise en Forme des Matériaux, CNRS UMR 7635, PSL Research University, CS 10207 rue Claude Daunesse, CEDEX, 06904 Sophia Antipolis, France; rudy.valette@mines-paristech.fr

**Keywords:** molecular architecture, long-chain branching, polybutylene succinate, biodegradable, size-exclusion chromatography, shear rheology, extensional rheology

## Abstract

The molecular architecture and rheological behavior of linear and branched polybutylene succinate blends have been investigated using size-exclusion chromatography, small-amplitude oscillatory shear and extensional rheometry, in view of their processing using cast and blown extrusion. Dynamic viscoelastic properties indicate that a higher branched polybutylene succinate amount in the blend increases the relaxation time due to an increased long-chain branching degree. Branched polybutylene succinate exhibits pronounced strain hardening under uniaxial elongation, which is known to improve processability. Under extensional flow, the 50/50 wt % blend exhibits the same behavior as linear polybutylene succinate.

## 1. Introduction

Nowadays, polymeric packaging materials are mostly based on non-renewable resources. However, due to growth of environmental issues, it becomes more and more obvious that alternatives must be considered to replace these conventional petroleum-based polymers by either biodegradable (or compostable) and/or bio-based polymers. For decades, the mostly used polymer matrices for packaging purposes have belonged to the polyolefin family (polyethylene (PE), polypropylene (PP)). The reasons for these polyolefins to hold such a monopoly are quite clear: they exhibit a very good processability due to their large operating window and thermal stability, they are relatively cheap (around 1–2 euros per kilogram depending on the properties), and a tremendous choice of tailored properties is available (e.g., improved thermal stability, various molecular features with controlled rheological properties…). 

The blow and cast film extrusion processes used to produce thin films for packaging purposes require high melt strength/elasticity and high viscosity polymer in order to avoid processing instabilities such as bubble instabilities in the case of blown-film extrusion [1,2]. Long-chain branched (LCB) structures have already demonstrated their efficiency in improving the melt strength compared to the linear structures. Indeed, LCB are known to exhibit strain hardening under elongational flow, which is required to obtain a homogeneous deformation during the stretching stage in the blown-film extrusion [3,4]. Beyond their impact on the rheological performances, LCB are also known to affect the crystallization and phase behavior in the solid state, leading to modified mechanical properties compared to their linear homolog.

Many studies on the influence of long-chain branching on the rheological properties of conventional polyolefins such as PE [1,3,5,6,7,8,9,10,11] or PP [4,12,13,14] have been carried out. In general, the authors agree on a definition of LCB (from a rheological point of view) as being branches whose weight is higher than the critical mass for entanglements, M_c_. The studies reveal that LCB with branches longer than twice *M_c_* have a significant impact on the processing properties [15] and have a strong effect on the viscosity, the elasticity, and the activation energy under small amplitude oscillatory shear flow. In addition, the effect of polydispersity on these parameters has been reported to be very similar to the one expected from LCB, leading to difficulties in separating the effects of different molecular features. Even if such relationships between molecular structures and resulting rheological properties have been extensively investigated, the lack of controlled molecular architectures in terms of degree of branching, branch length, and structure have seriously limited the type of polymers studied, being mostly restricted to some model polyolefins. Nonetheless, some studies have been performed on LCB biodegradable or bio-based polymer matrices. Very recently, Nouri et al. studied the impact of different types of branching on the rheological behavior under the extensional flow of poly (lactic acid) (PLA) [16,17]. They showed that branched PLAs exhibit large strain hardening, but they did not conclude on the effect of the type of branching on the results. Although the low melt strength and viscosity of PLAs usually prevent them from being used in a wide range of thermoplastic processes such as blown extrusion [18,19,20,21], they demonstrated that the addition of some branches can greatly improve the processability of PLA. Meanwhile, another biopolyester has attracted interest in these past 15 years, namely polybutylene succinate (PBS) thanks to its biodegradability and thermomechanical properties close to PE and PP [22]. PBS exhibits balanced performances in terms of thermal and mechanical properties as well as a good thermoplastic processability. PBS is a highly crystalline (around 50%) aliphatic polyester with a melting point of 115 °C (similar to PE) and a tensile yield strength (in the case of unoriented samples) that can reach up to 30–35 MPa (comparable to PP) [23]. PBS is usually synthetized via polycondensation of succinic acid and 1,4 butanediol (BDO), and its monomer can also be derived from renewable resources [23].

Linear grades of PBS have been developed and commercialized over the years, but to our knowledge, only the company Showa Denko High Polymer (SDK) was commercializing one grade of LCB PBS. Unfortunately, SDK abandoned the production of PBS in 2016. 

LCB polymers in general, and in particular newly developed (bio)polymers, are more expensive than linear polymers due to the more complex synthesis or additional reactive extrusion step to obtain them. As for PP, blending linear and branched PBS is therefore interesting to improve the processability of linear polymer while decreasing the price of the polymer.

The very few studies that report on the rheological properties of PBS concern linear or branched PBS and especially never point out any relationships between their molecular structure and rheological behavior. To our knowledge, there are no published works dealing with the influence of blending linear and branched PBS on the rheological behavior of PBS. In addition, only a few studies concerned blends of polyolefins especially in extensional flow [3]. This study aims to provide a deep structural and rheological characterization of the long-chain branching (so-called BPBS) and linear (so-called LPBS) PBS and to understand the influence of their blending on linear and nonlinear viscoelastic properties. First, an analysis of the macromolecular structure of both BPBS and LPBS was carried out using size-exclusion chromatography; then, the impact of their blending on linear and nonlinear viscoelastic properties was analyzed. Finally, the relationship between their macromolecular structure and rheological properties has been discussed.

## 2. Experimental 

### 2.1. Materials and Processing

A linear and a branched polybutylene succinate respectively referred as LPBS and BPBS produced by Showa Denko High Polymer (Tokyo, Japan) and commercialized under the trade names Bionolle 1001MD™ and Bionolle 1903MD™ (sourced through Sojitz Europe Plc, Paris branch, Paris, France), respectively, were chosen in this study. Since LPBS is obtained by the polycondensation of bifunctionnal monomers, the structure produced no longer contains any LCB neither SCB. On the other hand, BPBS results from the copolymerization of butylene succinate monomer with an additional monomer including multifunctional groups, leading to branched structures [24]. BPBS and LPBS properties (density ρ, Melt Flow Rate MFR, melting temperature *T_m_*, and architecture) according to the supplier datasheet are given in Table 1.

Blending and sample preparation were performed with a laboratory-scale extruder Polylab system (Thermo Fisher Scientific, Waltham, MA, USA) composed of a HAAKE RheoDrive4 motor unit/torque rheometer (Thermo Fisher Scientific, Waltham, MA, USA) with three thermoregulated zones coupled with a HAAKE Rheomex 19/25 OS single screw extruder (Thermo Fisher Scientific, Waltham, MA, USA). The extruder was equipped with a fish-tail designed die (270 mm width, 0.45 mm die gap) in order to obtain film samples. The extruder temperature zones were fixed at 240–235–230 °C and the die was fixed at 180 °C. The screw speed was kept constant at 80 rpm, 4.08 $\dot{m}$ (kg·h^−1^).

In addition to the two pure polymers, three BPBS/LPBS blends (20/80 wt %, 50/50 wt %, and 80/20 wt % BPBS/LPBS) were dry-mixed and melt-compounded in the extruder. PBS is known to be sensitive to water or residual carboxylic acids terminals [23], which can lead to degradation issues (i.e., hydrolysis) during processing; all materials were dried at 80 °C during 4 h in a vacuum oven prior to extrusion.

### 2.2. Characterizations

#### 2.2.1. Molecular Architecture Analysis

Triple detection size exclusion chromatography (SEC) was used to determine both the absolute molecular weight and level of branching of the blends. In the case of the use of a Low-Angle Laser Light Scattering detector (LALLS) or a Right-Angle Laser Light Scattering detector (RALLS) with a viscometer detector, both the molecular weight and intrinsic viscosity (and their distribution) of each fraction of the eluted polymer can be determined.

In this study, SEC measurements were carried out on a chromatograph composed of a Waters 515 HPLC pump with PLgel Mixed-C 5 µm 600 mm column set (AGILENT TECHNOLOGIES Inc., Santa Clara, CA, USA), a Waters 410 differential refractometer (DRI) sourced from AGILENT TECHNOLOGIES Inc. (Santa Clara, CA, USA), a Viscotek T60A dual detector (Right-Angle Laser Light Scattering detector at λ = 670 nm, RALLS, and a differential viscometer, IV-DP), sourced from Malvern Panalytical Ltd. (Malvern, UK).

Pellets were dissolved in chloroform to obtain a sample concentration of 20 mg/mL and solutions were filtered by a polytetrafluoroethylene (PTFE) membrane filter with 0.2 µm pores prior to SEC measurements. Chloroform was chosen as an eluent because of its good solubility and differential refractive index for polyesters (around 0.06 for PBS, calculated value). The mobile phase was CHCl_3_ at a flow rate 1.0 mL/min. Five times the injection volume of 20 µL was used in order to avoid a dead volume effect. The detector temperature was 35 °C and the column temperature was 20 °C. Universal calibration was performed using a linear polystyrene with a narrow polydispersity and an average M_w_ of 19.760 g/mol as a standard. All analyses were carried out on Viscotek software OmniSEC^®^ (v4.6), sourced from Malvern Panalytical Ltd. (Malvern, UK).

#### 2.2.2. Small Amplitude Oscillatory Shear Rheometry/Linear Viscoelasticity

Dynamical rheological measurements of LPBS, BPBS, and their blends were performed using a rheometer ARES (TA Instruments, New Castle, DE, USA) equipped with a 25 mm parallel plates geometry in oscillatory shear and steady mode at 150, 170, and 190 °C. Stacked films were used to obtain a 1.5 mm thick sample and then cut into a 25 mm diameter disk. Preliminary time sweeps were conducted at *γ* = 5% to check if any degradation occurred during the test. A slight decrease of complex modulus was observed for the LPBS at 190 °C, which was probably due to thermal degradation, after a few minutes. Then, measurements were conducted with three points per decade in order to shorten the test and prevent samples from degradation during the test. The strain value, for all experiments, was set to 5% (small amplitude), which was checked to keep all measurements in the linear regime. Then, frequency sweep measurements were performed, within the frequency range from 100 to 0.01 rad. s^−1^. During loading, the gap was progressively set to 0.8 mm, and a varying delay was applied to the polymer to reach equilibrium (zero normal force) before starting each test. All measurements were reproduced twice and were well reproducible. The viscoelastic parameters, namely, storage modulus (*G*′), loss modulus (*G*″), complex viscosity (η*), and loss angle (*δ*) were calculated by using TA Orchestrator^®^ software, sourced from TA Instruments (New Castle, DE, USA).

#### 2.2.3. Steady Shear Rheometry

Additional steady shear viscosity measurements were performed at 150, 170, and 190 °C, within the range of shear rate from 0.01 to 10 s^−1^, in order to check consistency with oscillatory measurements and access to lower shear rates.

#### 2.2.4. Elongational Viscosity Measurements

Elongational viscosity was measured using the TA Extensional Viscosity Fixture (EVF) of the ARES rotational rheometer (both sourced from TA Instruments, New Castle, DE, USA). Films of 10 mm width and 18 mm length were directly cut into the center of the width of the extruded film (to avoid edge bead effects and hence an inhomogeneous thickness of the films across their width) and stacked to obtain the 0.7 mm targeted thickness required to reach a sufficient level of force. Careful attention was paid to avoid slippage within the stack when clamped on each rotating drum. The tests were performed at constant strain rates of 0.01, 0.1, 1, and 10 s^−1^ and at 150 °C to prevent the samples from sagging. At least two trials were performed at each extension rate for each sample to ensure reproducibility of the results.

## 3. Results and Discussion

### 3.1. Analysis of the Macromolecular Architecture of Linear and Branched PBS

The values of weight- and number-average molecular weights (*M_w_* and *M_n_* respectively), polydispersity index (PI as the *M_w_*/*M_n_* ratio), intrinsic viscosity [*η*] and Mark Houwink Sakurada (MHS) parameters related to each polymer are listed in Table 2. The mean value of the radius of gyration (*r_g_*) was directly calculated by the software from the measured value of the hydrodynamic radius *r_h_* thanks to the Fox–Flory relationship [25].

**Figure 1:**
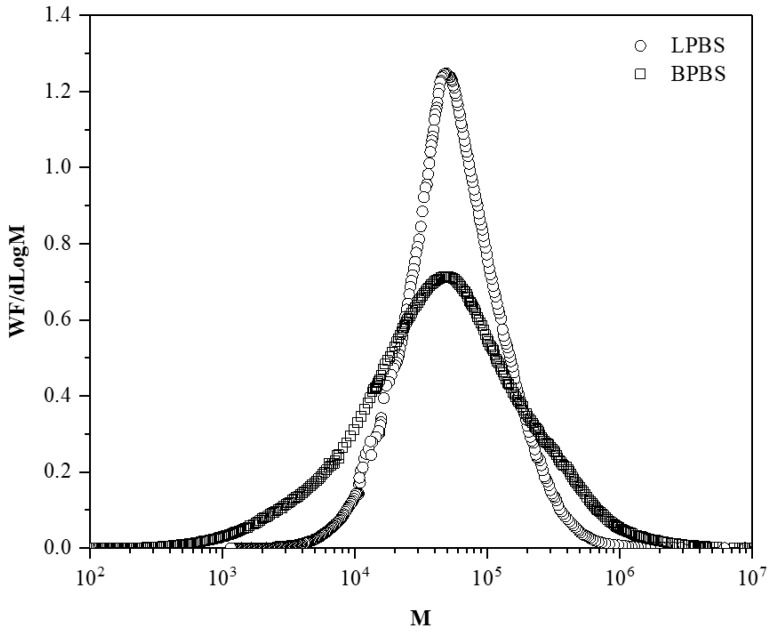
Molecular weight distributions of BPBS (□) and LPBS (○).

Molecular weight distributions of LPBS and BPBS samples are illustrated in Figure 1 and show the relatively narrow distribution of the linear PBS (LPBS) compared to the broader distribution of the branched PBS (BPBS) that is in agreement with the polydispersity values. The high polydispersity index and broad molecular weight distribution of BPBS is a first evidence of the presence of LCB in this PBS grade [26]. Both samples exhibit a log-Gaussian molar mass monomodal distribution. The intrinsic viscosities of LPBS and BPBS, denoted [*η_LPBS_*] and [*η_BPBS_*], respectively, were directly measured from the viscosity detector for each eluted fraction of polymer. In a semi-logarithmic graph, MHS parameters were determined from the slope and intercept of the intrinsic viscosity as a function of molecular weight:

(1) $$\left[\eta \right]=K\times M_{\nu }^{a}$$

where *K* and *a* are dependent on the considered polymer–solvent system and on the measurement temperature.

The exponent *a* gives information on the conformation of the polymer chains in the solvent. A value between 0 and 0.5 is generally obtained for branched chains, and a value comprised between 0.5 and 0.8 for is obtained for flexible chains [25]. The MHS parameters of PBS were reported only once by Garin et al. on an in-house synthetized linear PBS characterized by SEC coupled with multi-angle laser light scattering (SEC-MALLS). They found an average value of *a* = 0.71 (+/−0.1) and *K* = 39.94 × 10^−5^ (+/−6.31 × 10^−5^) dL·g^−1^ [27], which are in total agreement with our values for LPBS. However, to the best of our knowledge, no study has previously reported the MHS parameters for long-chain branched PBS. For BPBS, we found a much higher value of *K* = 325 × 10^−5^ dL·g^−1^ and a lower value of *a* = 0.528, indicating a lower flexibility of polymer chains related to branching. In their study on the role of the architecture on the conformation in dilute solution of in-house synthetized polystyrenes (PS), Kharchenko et al. proposed a deep characterization of linear, star, and hyperbranched PS. For the same branching density, they found that a star PS exhibited a lower value (*a* = 0.68) and even much lower value for hyperbranched PS (*a* = 0.39) compared to the linear counterpar of equivalent molecular weight.

**Figure 2:**
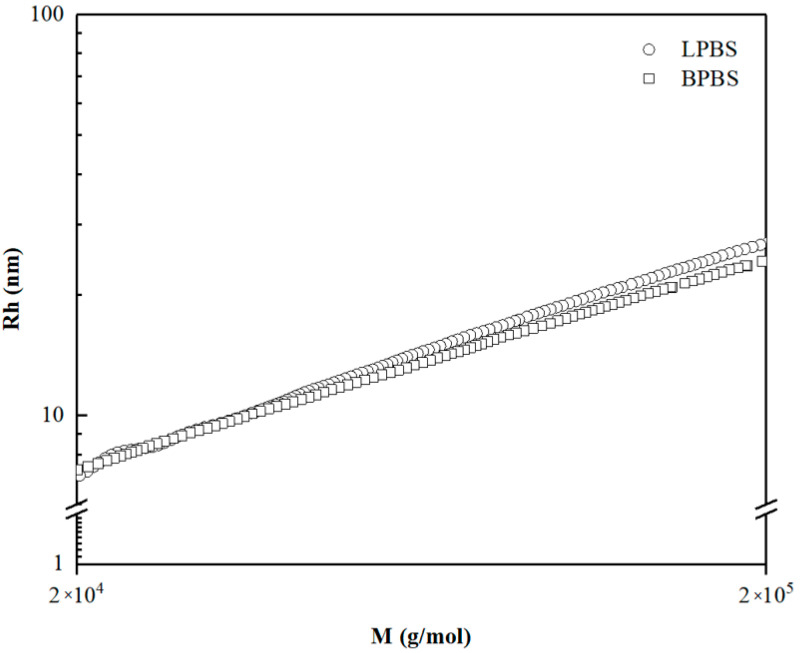
Log-log plot of hydrodynamic radius of BPBS (□) and LPBS (○).

Figure 2 shows the hydrodynamic radius of LPBS and BPBS as a function of molecular weight *M*. At low molecular weight, BPBS and LPBS exhibit the same value of hydrodynamic radius, meaning that the macromolecules below 40,000 g/mol are mostly linear. Above 40,000 g/mol, the radius of BPBS fall below that of LPBS, as expected by the lower volume occupied by the coil of a branched chain which is more compact than a linear one of the same molar mass [4]. Thus, the continuous decrease of the radius depicts the increase of LCB as the molecular weight increases.

**Figure 3:**
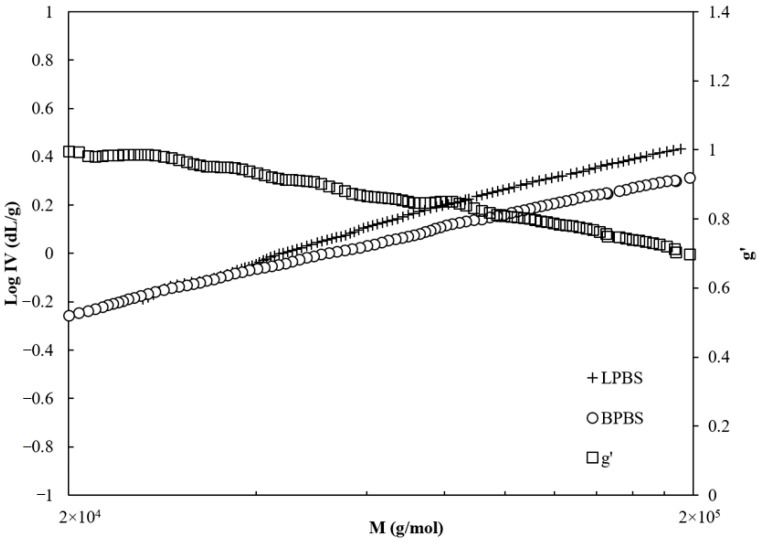
Intrinsic viscosity of LPBS (□) and BPBS (◇) and $g^{\prime }$ the viscosity branching index (○), dotted line representing Mark Houwink Sakurada (MHS) semi-logarithmic line.

Consequently, to the reduction of the hydrodynamic radius in this range of molecular weight (i.e., >40,000 g/mol), the intrinsic viscosity of BPBS is lower than that of LPBS, as shown in Figure 3. At the same time, the viscosity branching index ($g^{\prime }$) (given by Equation (2)) decreases almost monotonically from 0.9 ($g^{\prime }$ would be equal to 1 for linear chains) to 0.65 on the range of molecular weights accordingly with the BPBS decreasing viscosity.

(2) $$g^{\prime }={\frac{{\left[\eta \right]}_{b}}{{\left[\eta \right]}_{l}}|}_{=M}$$

where [*η*] is the intrinsic viscosity and the subscripts *b* and *l* correspond to the branched and linear counterpart, respectively, which are considered at the same molecular weight.

The decrease of viscosity branching index indicates that the chains are mostly linear below 40,000 g/mol and that higher molecular weight molecules have a larger branching degree.

Assuming that the BPBS is randomly branched, which is the case for most branched polyesters in nature [28], a Zimm–Stockmayer model for randomly distributed branch points per molecule with a random distribution of branch length (polydispersed) tri-functional polymers [29] (Equation (3)) was used to determine the branching characteristics of the polymer.

(3) $$g=\frac{6}{B_{w}}\;\left[\frac{1}{2}{\left(\frac{2+B_{w}}{B_{w}}\right)}^{\frac{1}{2}}ln\left(\frac{{\left(2+B_{w}\right)}^{\frac{1}{2}}+B_{w}{}^{\frac{1}{2}}}{{\left(2+B_{w}\right)}^{\frac{1}{2}}-B_{w}{}^{\frac{1}{2}}}\right)-1\right]$$

The former relationship links the branching index (g) to the weight-average number of branch points per molecule (*B_w_*) and the LCB frequency (λ) (average number of branch points per 1000 monomers) given by:

(4) $$\lambda =\frac{1000B_{w}M_{0}}{M_{n}}$$

where *M*_0_ is the molecular weight of a monomer and *M_n_* is the number-average molecular weight. For PBS, *M*_0_ = 172 g/mol.

In the present study, only the viscosity branching index $g^{\prime }$ was determined. The viscosity branching index and the branching index g are related through *ε* known as the shielding factor (sometimes called the drainage factor) equal to 3/2 in the non-draining case. This factor is known to be dependent on the branching structure and has shown to be equal to 0.5 for star polymers while *ε* is closer to 1.5 for comb-shaped structures [30]:

(5) $$g^{\prime }=g^{\varepsilon }=g^{\frac{3}{2}}$$

**Figure 4:**
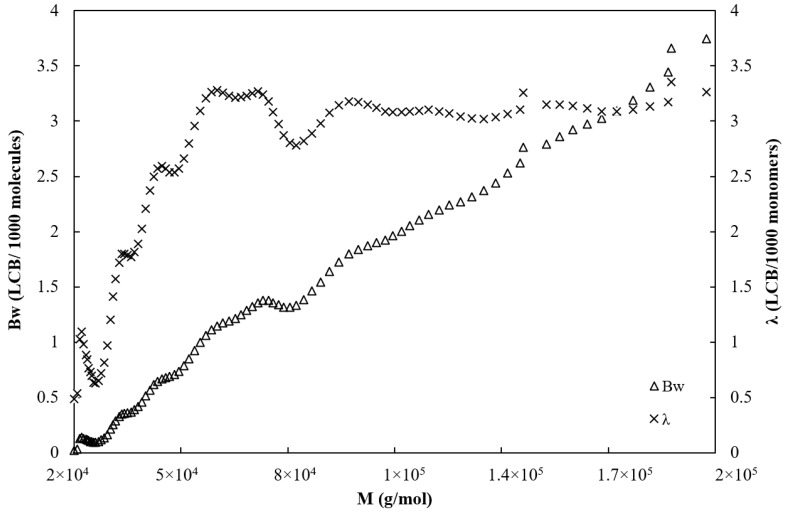
Average number of branch points per molecule *B_w_* (Δ) and long-chain branched (LCB) frequency per 1000 monomer units *λ* (×) for BPBS.

Figure 4 shows the average number of branch points per molecule *B_w_* and the LCB frequency λ plotted versus the molecular weight. The number of branches is ranged from 0 and 0.5 between 20,000 and 40,000 g/mol and starts to grow sharply above 40,000 g/mol from 0.5 to 3.7 at 200,000 g/mol. 

Meanwhile, the branching frequency increases sharply from 0.5 LCB per 1000 units to approximately 3 LCB per 1000 units in the range of molecular weight comprised between 20,000 and 60,000 g/mol and then remains constant at 3 LCB/1000 units on the range of 60,000 and 200,000 g/mol. 

Values of the average viscosity branching index, average number of LCB per molecule, and LCB frequency calculated by OmniSEC software are given in Table 3.

Only very scarce studies were carried out on the characterization of long-chain branched polybutylene succinate. Although Wang et al. [31] studied the synthesis, characterization, and properties of long-chain branched PBS, they did not evaluate the branching characteristics and only determined the conventional molecular features (*M_w_*, *M_n_*, PI) by the use of conventional calibration (leading to possible misinterpretation of *M_w_*). Kim et al. [32] used SEC-MALLS to determine the molecular characteristics of in-house synthetized branched PBS in chloroform. Unfortunately, they did not report any branching characteristics nor MHS coefficients. Two decades ago, Yoshikawa et al. performed SEC coupled with multi-angle laser light scattering (MALLS) detector on the same polymer grades used in this study [24]. The authors did not report the distribution of the number of LCB per molecule as a function of molecular weight and did not report the branching frequency.

They only give a mean value of the branching index as a function of the molecular weight calculated from pretty scattered data. They found an average number of branch points close to 2 LCB per molecule, which is consistent with our results and could confirm the shielding factor hypothesis we made. Finally, with a mean value of 2 LCB per molecule, Yoshikawa et al. suggested that the branching architecture could be an H-shaped molecule.

Focusing on the studies reporting the branching structure of polyolefins, the rheological behavior of the blends of linear and branched polymers depended heavily on the amount of LCB, on the molecular weight, on the type of branching (or topology), and on the relative composition of the mixture. 

Stange et al. studied the impact of blending linear and long-chain branched PP on the shear and elongational flow behavior [4]. They showed that increasing the amount of LCB in the LCB-PP/linear PP blend resulted in a more pronounced shear thinning behavior, which is beneficial for processing. This effect was attributed to both the higher polydispersity and the increasing amount of LCB with the addition of LCB-PP in the blend. The type of architecture has also been proven to greatly influence the rheological behavior. For instance, the Zero-Shear rate Viscosity (ZSV) of long-chain branched PE with a moderate degree of star-like molecular structure has been shown to be enhanced over that of its linear counterpart, whereas a highly tree-like long-chain branched one exhibits a lower ZSV than its linear homologue [4].

Despite the difficulty of understanding the relationships between the molecular architecture and the resulting rheological properties, especially regarding the commercial nature and the impact of blending architectures, we show, in the following, a relationship with the rheological characterization of these architectures and blends.

### 3.2. Impact of Blending Linear and Branched PBS on Rheological Behavior

#### 3.2.1. Rheometry under Oscillatory Shear Flow

**Figure 5:**
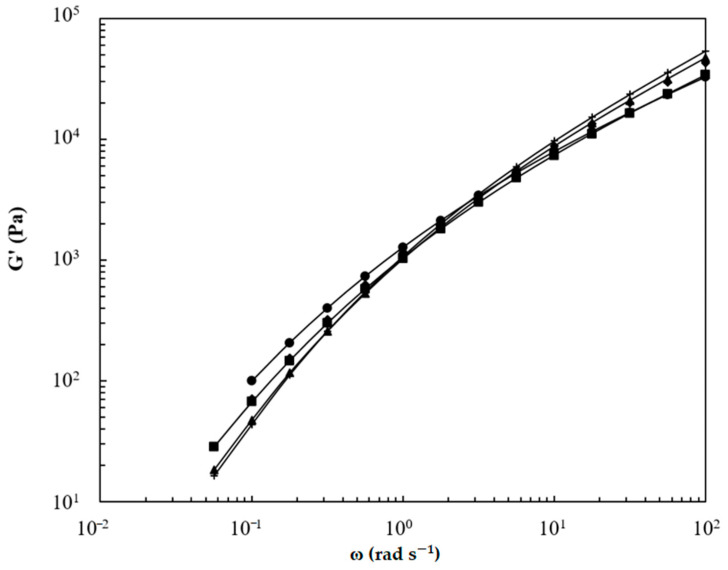
Storage modulus of BPBS (•), LPBS (+), and BPBS/LPBS blends (■) 80/20%, (◆) 50/50%, (▲) 20/80% at 150 °C.

**Figure 6:**
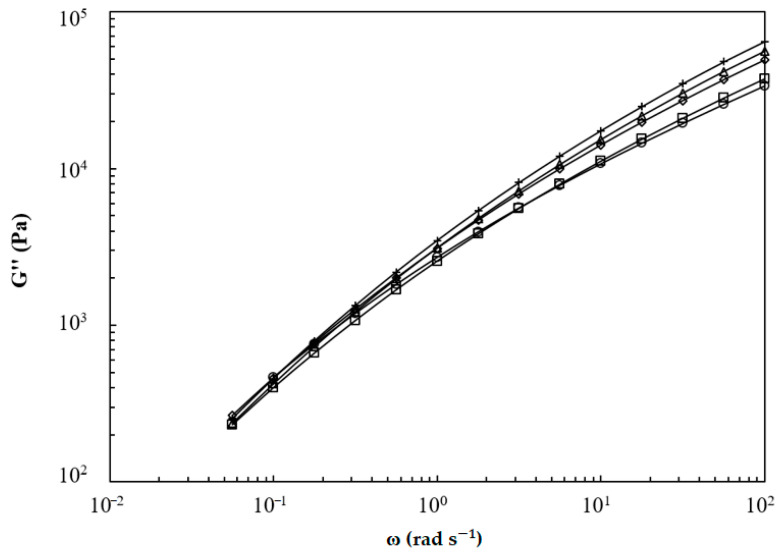
Loss modulus of BPBS (○), LPBS (+) and BPBS/LPBS blends (□) 80/20%, (◇) 50/50%, (Δ) 20/80% at 150 °C.

Storage (*G*′) and loss moduli (*G*″) of LPBS, BPBS, and their blends at 150 °C are illustrated in Figure 5 and Figure 6, respectively. The loss modulus is found to dominate the flow of every composition on the entire range of frequency tested (i.e., no crossover is highlighted). At low frequencies, an enhancement of storage modulus is found for BPBS compared to LPBS and the blends lie in between the pure components, while the opposite trend is observed at high frequencies. No major difference of *G*″ according to the composition is highlighted at low shear rates, while at high shear rates, the LPBS shows the highest loss modulus and BPBS shows the lowest.

The storage and loss moduli slopes at low frequency for LPBS, BPBS, and their blends at 150 °C are listed in Table 4. As expected, the terminal zone was almost reached for LPBS, especially for the *G*″ slope. The value of 1.67 for the *G*′ slope (power law exponent) indicates that there are long (>10 s) relaxation processes, which is probably due to the high molecular mass tail of the distribution (see Figure 1). When the amount of BPBS increases, the slope of *G*′ decreases from 1.67 for the LPBS to 1.21 for the BPBS and the slope of *G*″ decreases from 0.99 for the LPBS to 0.82 for the BPBS. Clearly, a lower frequency (longer time) is needed for the LCB polymer to reach the terminal zone, and increasing the BPBS weight fraction results in a terminal zone reached at lower frequency. Wang et al. [33] reported the same trend when blending linear and branched PLA. Increasing the PLA branched weight fraction resulted in a higher deviation from the linear terminal relaxation zone. They argued that at low frequency, where only the high relaxation times contribute to the viscoelastic behavior, the behavior exhibited by the blends is ascribed to the presence of LCB. Since BPBS and LPBS have a similar weight-average molecular weight, this result confirms the effect of LCB on the increase in relaxation times. Indeed, LCB favor relaxation by arm fluctuation, which is associated with much larger relaxation times than the relaxation by reptation encountered in linear chains [34]. Moreover, as BPBS is more polydispersed than LPBS, the broadening of relaxation times shifts the terminal zone down to lower frequencies. Furthermore, it is also noted that the rubbery region has not been reached, whatever the composition of the mixture considered. As a matter of fact, it is also well known that LCB and polydispersity lead both to similar effects in broadening the transition region between the terminal zone and the plateau modulus. Therefore, in the frequency range tested, only the behavior in the transition zone can be noticed. 

At high frequency (ω = 100 rad·s^−1^), *G*′ and *G*″ decrease when the amount of BPBS increases. Conversely, at low frequency (ω = 0.05 s^−1^), the incorporation of BPBS leads to an enhancement of *G*′ while *G*″ remains almost constant. The low-frequency region is known to be the most sensitive to molecular features [15,33]. Therefore, the enhancement of *G*′ in the low-frequency region is attributed to the longer relaxation time ascribed to the LCB. Therefore, the more LCB, the higher the *G*′ enhancement, and thus, the higher elasticity and melt strength (i.e., a more notable solid-like behavior). 

The ZSV of LPBS, BPBS, and their blends was estimated from steady-state experiments carried out on the shear rate range 0.01 to 1 s^−1^ at 150 °C. At low shear rates, the complex viscosity |η*| (not shown) exhibits a constant value for all blends and pure polymers. The BPBS exhibits a slightly higher ZSV than LPBS (4569 Pa.s for LPBS vs. 5555 Pa s for BPBS). Given that the weight-average molecular weight is considered similar between the two neat components and that the ZSV is known to be insensitive to the molecular weight distribution, it is suggested that this improvement in elasticity is due to LCB.

#### 3.2.2. Cox–Merz Rule and Carreau–Yasuda Model

**Figure 7:**
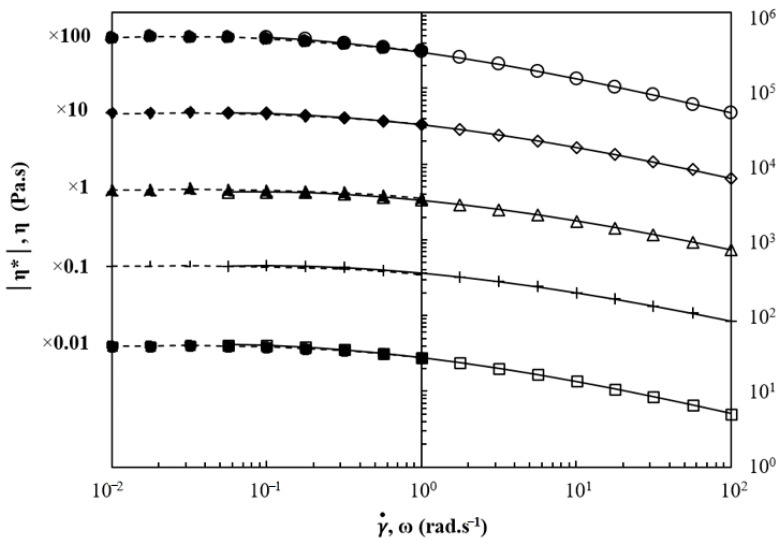
Cox–Merz rule and Carreau–Yasuda model applied to BPBS (○), LPBS (+), and BPBS/LPBS blends (□) 80/20% (◇) 50/50%, and (Δ) 20/80% at 150 °C (steady shear full symbols, dashed line; oscillatory shear open symbols, Carreau–Yasuda fit solid line).

The steady-state *η* viscosity and complex viscosity |η*| curves were superposed following the Cox–Merz rule [35], and the Carreau–Yasuda [36] model (Equation (6)) was fitted on each curve (as illustrated in Figure 7):

(7) $$\left|\eta^{*}\left(\omega \right)\right|=\eta_{0}{\left[1+{\left(\lambda \omega \right)}^{\alpha }\right]}^{\frac{\left(n-1\right)}{\alpha }}$$

where *η*_0_ is the ZSV, *λ* is the characteristic relaxation time, ω is the angular frequency, *n* is the flow index reflecting shear-thinning, and α is the parameter describing the width of the transition between the terminal and the shear thinning regimes of slope (*n* − 1). 

For sake of clarity, every piece of experimental data was shifted by one or two decades higher or lower according to its rank when not shifted. The superposition seems to hold for every blend. No major deviation is clearly seen from the Cox–Merz rule, but the log-log plot often hides small deviations. As a matter of fact, a slight deviation is observed for the BPBS. In their study on the consequence of blending PLA of different chain architectures, Lehermeier and Dorgan [37] reported the same lack of superposition between steady shear viscosity and dynamic oscillatory (small amplitude) shear viscosity. Better agreement with the Cox–Merz rule is found for linear architecture than for long-chain branched polymers [35]. Utracki and Gendron [38] in their study on the behavior of LDPE (i.e., LCB Low-Density Polyethylene) during extrusion reported a similar breakdown that they suggested to be related to the existence of strain-hardening in elongational flow. This latter suggestion leads to think that the LCB is the cause of the lack of superposition. 

The resulting parameters of the Carreau–Yasuda fit are summarized in Table 5. The fitted data of zero-shear rate viscosity show a good agreement with the experimental ones for all blends. The same trend as that reported above can be depicted from the fitted data concerning the behavior of ZSV as a function of the PBS weight fraction.

It can be seen from Table 5 that increasing BPBS in LPBS results in an increase of the characteristic relaxation time and a progressive decrease of the shear thinning index *n*. Nevertheless, the 20/80% BPBS/LPBS blend exhibits the same shear thinning than the LPBS and a close ZSV, which seems to indicate that adding 20% of BPBS in LPBS has no strong effect on the rheological behavior under shear flow (except from the slight increase of relaxation time).

The increase in relaxation time and shear thinning of branched polymers compared to their linear counterparts has already been reported in the case of PE [39], PP [4], and PLA [33]. The progressive increase of the relaxation time below 80% of BPBS in the blend suggests a good correlation between the relaxation time and the amount of branching (since the type and length should be similar in all blends) and supports the theory of miscibility at those BPBS weight fractions. Similar findings were also reported by Liu et al. [10] for LDPE/LLDPE blends. In addition, the improvement of shear thinning index can also be attributed to the large polydispersity of BPBS. In fact, the polydispersity affects the shear thinning index in the same way that LCB does, and separating the coupled effects appears as a complicated task. Hence, the shear thinning index includes both the effect of LCB and of the molecular weight distribution.

The relaxation spectrum gives valuable information on the distribution of relaxation times and therefore on the structure of the blend. The spectrum in its discrete form is constructed from the Generalized Maxwell Model (GMM) with seven elements, according to Laun’s method [40].

The spectrum of the miscible blends is expected to result in a single peak with a smooth transition from one pure component to another [8]. The sum of the resulting relaxation strengths $G_{i}$ is assimilated to the plateau modulus $G_{N}^{0}$ [15] as:

(7) $$G_{N}^{0}=\sum^{\;}G_{i}$$

In addition, the discrete constants given by the general Maxwell model allows the estimation of the zero-shear rate viscosity *η*_0_ and the mean relaxation time $\bar{\lambda }$ according to Equations (8) and (9).

(8) $$\eta_{0}=\overset{N}{\underset{i}{\sum }}G_{i}\lambda_{i}$$

(9) $$\bar{\lambda }=\frac{\sum^{\;}\lambda_{i}^{2}G_{i}}{\sum^{\;}\lambda_{i}G_{i}}$$

The results of relaxation strength normalized by the instantaneous modulus (weighed relaxation strength *ω_i_*) are summarized in Table 6.

On the frequency range tested (i.e., where the plateau and terminal zones are missing), we can observe a clear increase of the weighted relaxation strengths with the BPBS weight fraction at higher relaxation times. Given that the weight-average molecular weights of the LPBS and BPBS are similar, it can be suggested that the increase of the BPBS amount in the blend is responsible for the weighted strengths moduli to shift to longer relaxation times. Furthermore, the broadening of the relaxation spectrum together with the apparition of a shoulder for higher BPBS-rich blends for the higher relaxation times leads to the same conclusion that the weighted relaxation strengths are more significant at longer times because of the increased number of LCB in the blend [12].

Then, the relaxation modulus and relaxation time constants {*G_i_*, *λ_i_*} were used as fitting parameters (in a nonlinear mean square minimization procedure) so that $G_{N}^{0}$ and *η*_0_ were estimated more accurately (Equations (7) and (8)). The results of the second fitting procedure in term of $G_{N}^{0}$, $\bar{\lambda }$, and *η*_0_ are listed in Table 7.

The estimated plateau modulus is found to be in the same range as other polyesters such as Polylactic acid (PLA) ($G_{N}^{0}$ = 0.90 × 10^5^ Pa for L-PLA and 2 × 10^5^ Pa for D,L-PLA [41]) or Polyhydroxybutyrate-co-3-hydroxyvalerate (PHBV) (*G_N_*^0^ = 1.53–1.97 × 10^5^ Pa) [41]) and even Polyethylene terephthalate (PET) ($G_{N}^{0}$ = 0.72–1.06 × 10^5^ Pa [42]). Moreover, the plateau modulus decreased with increasing BPBS weight fraction in the blend due to LCB. To our knowledge, the PBS plateau modulus value was reported only once before by Garin et al. [27] (mean value of $G_{N}^{0}$ = 1.5 × 10^5^ Pa) and is in total agreement with the value reported in this paper.

#### 3.2.3. Extensional Viscosity

Up to now, all investigated viscoelastic properties were measured under oscillatory shear flow and especially at low deformation (*γ* = 5%). However, these small deformations and deformation rates are quite far from those experienced during the cast film extrusion process used in this study. Therefore, elongational experiments may provide relevant information about the structural features of molecules that are not revealed by shear data and that actually influence the final properties of the polymer. The latter being not as effective as extensional flow in generating a significant stretching of the chains.

In the case of a Newtonian flow, Trouton [43] firstly noted that the elongational viscosity *η_E_* equals three times the zero-shear rate viscosity. This quantity is usually referred as Trouton ratio (Equation (10)).

(10) $$\eta_{E}\left(\dot{\varepsilon }\right)=3\eta_{0}\eta_{E}\left(\dot{\varepsilon }\right)=3\eta_{0}$$

It is of universal practice to compare the results of elongational flow experiments (the nonlinear material viscosity function) $\eta_{E}^{+}\left(t,\;\dot{\varepsilon }\right)\;$ with the linear response given by:

(11) $$\eta_{E}^{+}=3\;\overset{N}{\underset{i=1}{\sum }}G_{i}\lambda_{i}\;\left(1-e^{\frac{-t}{\lambda_{i}}}\right)\eta_{E}^{+}=\eta_{E}-3\;\overset{N}{\underset{i=1}{\sum }}G_{i}\lambda_{i}\;e^{\left(\frac{-t}{\lambda_{i}}\right)}$$

where $\left\{G_{i},\lambda_{i}\right\}$ are the previously mentioned GMM sets of parameters. 

In this context, if the datasets are accurately obtained, then the nonlinear response should agree with the linear one at short times and low strain rates. In addition, the deviation of the nonlinear response from the linear one (also called linear viscoelastic envelope, LVE) is used to qualify the behavior of the material under elongational flow. If the material under elongational flow exhibits a sudden rise deviating above the LVE, the melt is said to be strain hardening, which improves processability, whereas if it drops below the LVE curve, it is said to be strain softening.

Strain hardening has been shown for several LCB polymers such as PP [13], PS [44], and even PLA [45]. Stange et al. [4] showed that even a small amount of LCB-PP (<10 wt %) in blend with linear PP was responsible for strain-hardening behavior at all strain rates tested (from 0.01 to 1 s^−1^).

**Figure 8:**
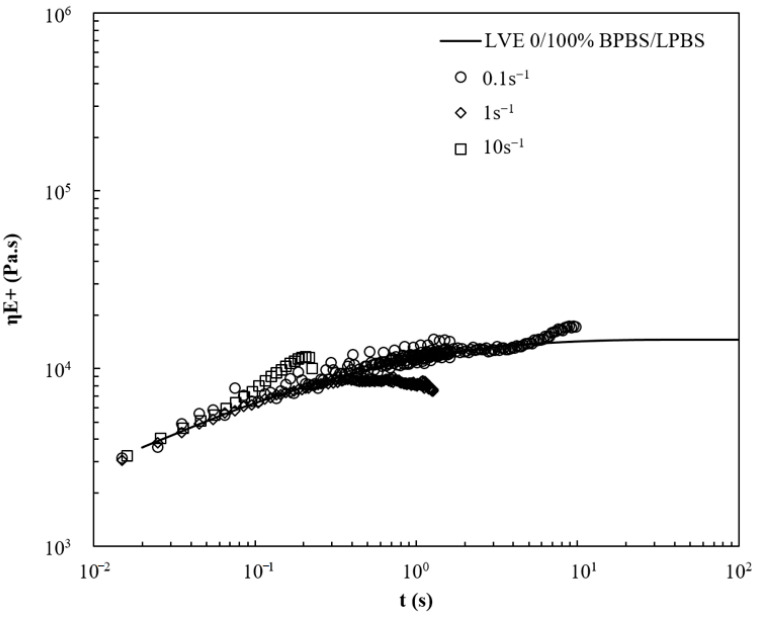
Viscosity curves under extensional flow at various strain rates for LPBS at 150 °C.

**Figure 9:**
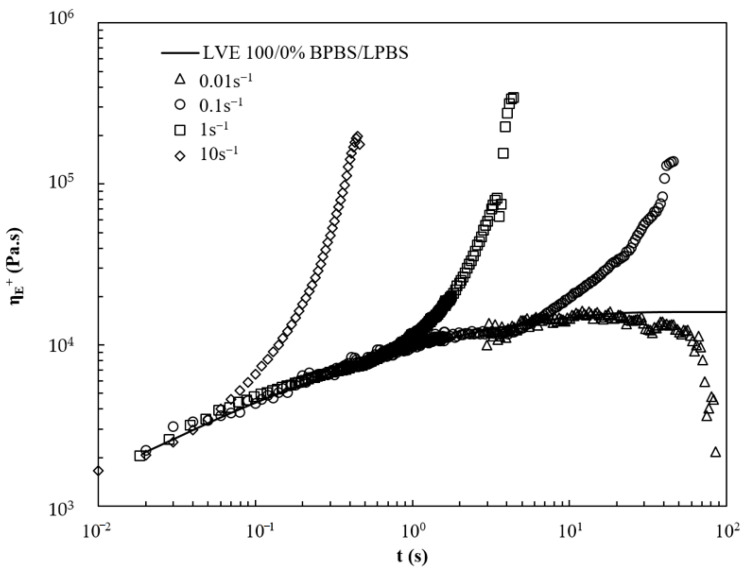
Viscosity curves under extensional flow at various strain rates for BPBS at 150 °C.

**Figure 10:**
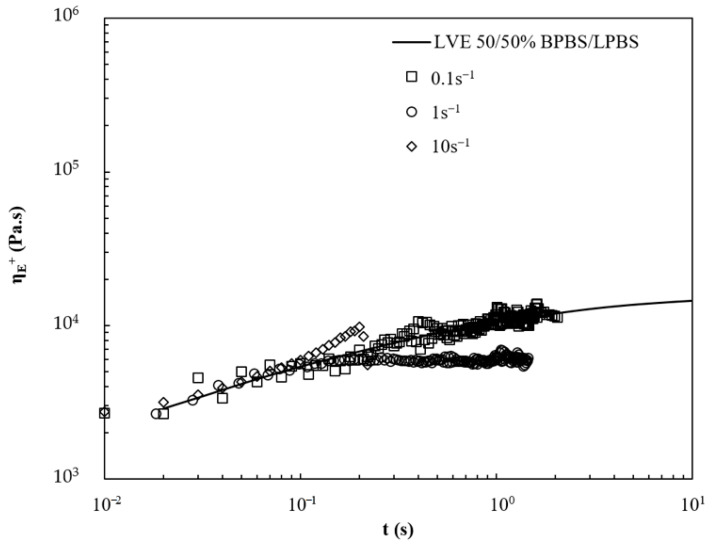
Viscosity curves under extensional flow at various strain rates for 50/50% BPBS/LPBS at 150 °C.

The extensional viscosity curves versus time of LPBS, BPBS, and 50/50% BPBS/LPBS at 150 °C and various strain rates ($\dot{\varepsilon }$ = 0.01 s^−1^, 0.1 s^−1^, 1 s^−1^ and 10 s^−1^) are presented in Figure 8, Figure 9 and Figure 10. For LPBS (Figure 8), at $\dot{\varepsilon }$ = 0.1 s^−1^, LPBS acts as a Newtonian fluid falling on the LVE. Above 4 s, the polymer exhibits slight strain hardening. Given that the average relaxation time $\bar{\lambda }$ (given in Table 7) of LPBS is 1.10 s, this strain hardening can be possibly due to a few of the longest chains (i.e., higher relaxation time), which remain stretched before the sample breaks. At $\dot{\varepsilon }$ = 1 s^−1^, the elongational viscosity curve deviates from the LVE exhibiting strain softening above 0.30 s. At this rate, the major part of the chains tends to disorient and relax due to high mobility. The strain-softening behavior of linear chains polymer is explained by a fast retraction dynamic, as occurs for shear thinning. Finally, slight strain hardening is observed for the highest stretching rate of $\dot{\varepsilon }$ = 10 s^−1^ from 0.06 s as the inverse Rouse (stretch) timescale of the chains is reached.

BPBS elongational viscosity (Figure 9) exhibits strain softening at $\dot{\varepsilon }$ = 0.01 s^−1^ due to the higher time of stretching compared to the average time of relaxation (given in Table 7). However, above these strain rates, the BPBS shows strain hardening with the level of strain hardening going up with the strain rates. In fact, LCB results in a longer relaxation time for BPBS compared to LPBS as mentioned earlier; therefore, when the strain rates increase, there are more and more chains extended accordingly to their high stretch relaxation time [34].

The 50/50% BPBS/LPBS blend in Figure 10 shows close behavior to that of LPBS. At the lower rate, it shows a linear response but exhibits strain softening at intermediate rate. At $\dot{\varepsilon }$ = 10 s^−1^, a slight strain hardening is observed. 

From these results of elongational viscosity, it can be suggested that BPBS strain hardening is related to the presence of LCB. The structure (number and length) of these LCB causes BPBS to exhibit strain hardening. Conversely, high strain rates are required to obtain slight strain hardening in LPBS. The 50/50% BPBS/LPBS blend behaves identical to LPBS. From this last observation, it can be suggested that either the LCB content is too low or the chain length is too small to produce significant strain hardening on the range of strain rates tested. Further characterizations of blends with higher BPBS content might reveal an increased strain hardening on this strain rates range.

## 4. Concluding Remarks

In this study, the rheological shear and elongational behavior of linear, long-chain branched PBS and their blends was investigated with further effort in understanding the impact of blending linear and LCB structures. An effort was made to deeply characterize the architecture of the commercial LCB PBS grade chosen for the purpose of this study. 

Molecular characterization confirmed that LPBS was purely linear and that BPBS exhibits approximately two branches per molecule (supposed H-shape). Although the number-average molecular weights were found to be similar for the two pure BPBS and LPBS, their polydispersity and architecture were found to be extensively different. These dissimilarities prevented us from separating the effect of each molecular feature on the rheological properties of pure components. It appears even more complex if considering their blends.

The discrete relaxation time spectrum based on a seven-mode Generalized Maxwell Model allowed us to focus the interpretation based on the highest relaxation time, for which we observed a clear increase of the weighted relaxation strengths when the BPBS weight fraction increases. 

However, if increasing the content of BPBS in the blend has only a marginal effect on the previous results under shear flow, it profoundly influences the transient elongational viscosity. 

Under elongational flow, the rheological response of LPBS and BPBS changed from shear thinning to strain hardening with the increase of strain rate. The BPBS showed a marked strain hardening at a lower strain rate compared to LPBS due to the higher relaxation time needed for its arm to disentangle. 

An incorporation of 50% of BPBS in LPBS did not significantly change the elongational response compared to LPBS. 

## Figures and Tables

**Table 1:** Bionolle™ branching polybutylene succinate (BPBS) and linear polybutylene succinate (LPBS) grades properties.

	BPBS	LPBS
*ρ* (g/cm^3^)	1.24	1.24
MFR (2.16 kg/190 °C)	4.5	1.5
*T_m_* (°C)	114–115	114–115
Topology	LCB	Linear

**Table 2:** Molecular characteristics of linear and branched PBS.

	*M_w_* (g/mol)	*M_n_* (g/mol)	PI	[*η*] (dL/g)	*r_g_* (nm)	MHS Parameters	
						*K* × 10^5^ (dL/g)	*a*
LPBS	123,500	37,800	3.3	1.37	14.7	42.4	0.725
BPBS	122,900	14,700	8.4	1.21	14.7	325	0.528

**Table 3:** Long-chain branching characteristics. Viscosity branching index, average LCB per molecule and average branching frequency per 1000 units for BPBS between 20,000 and 300,000 g/mol.

	$g^{\prime }$	B_w_ (LCB/Molecule)	λ (LCB/1000 Monomers)
**BPBS**	0.82	2.03	2.8

**Table 4:** Slopes (power law exponents) of storage and loss moduli for BPBS, LPBS and their blends at 150 °C.

BPBS/LPBS (wt %)	0/100	20/80	50/50	80/20	100/0
*G*′ slope	1.67	1.60	1.45	1.43	1.21
*G*″ slope	0.99	0.97	0.93	0.91	0.82

**Table 5:** Carreau–Yasuda parameters for LPBS, BPBS, and their blends at 150 °C.

BPBS/LPBS (wt %)	0/100	20/80	50/50	80/20	100/0
*η*_0_ [Pa s]	4569	4616	4855	4144	5555
*λ* [s]	0.69	1.04	1.24	1.35	2.21
*n*	0.61	0.61	0.59	0.58	0.55
*a*	0.90	0.89	0.88	0.92	0.89

**Table 6:** Weighted relaxation strengths from the Generalized Maxwell Model (GMM) for BPBS, LPBS, and their blends at 150 °C.

BPBS/LPBS (wt %)	0/100	20/80	50/50	80/20	100/0
$\lambda_{1}$ = 10^−5^ s	6.45 × 10^−10^	7.38 × 10^−10^	8.25 × 10^−10^	1.09 × 10^−9^	1.20 × 10^−9^
$\lambda_{2}$ = 10^−4^ s	1.54 × 10^−9^	1.76 × 10^−9^	1.97 × 10^−9^	2.61 × 10^−9^	2.87 × 10^−9^
$\lambda_{3}$ = 10^−3^ s	7.46 × 10^−1^	7.48 × 10^−1^	7.48 × 10^−1^	7.37 × 10^−1^	7.28 × 10^−1^
$\lambda_{4}$ = 10^−2^ s	2.06 × 10^−1^	2.03 × 10^−1^	1.98 × 10^−1^	2.03 × 10^−1^	2.02 × 10^−1^
$\lambda_{5}$ = 10^−1^ s	4.17 × 10^−2^	4.27 × 10^−2^	4.68 × 10^−2^	5 × 10 × 10^−2^	5.79 × 10^−2^
$\lambda_{6}$ = 1 s	5.45 × 10^−3^	5.90 × 10^−3^	7.39 × 10^−3^	8.82 × 10^−3^	1.15 × 10^−2^
$\lambda_{7}$ = 10 s	1.25 × 10^−4^	1.78 × 10^−4^	3.58 × 10^−4^	4.68 × 10^−4^	8.74 × 10^−4^

**Table 7:** Plateau modulus *G_N_*^0^, mean relaxation time $\bar{\lambda }$, and the zero-shear rate viscosity *η*_0_ of BPBS, LPBS, and their blends at 150 °C.

BPBS/LPBS (wt %)	0/100	20/80	50/50	80/20	100/0
$G_{N}^{0}$ (Pa)	1.99	1.75	1.57	1.20	1.15
$\bar{\lambda }$ (s)	1.10	1.36	2.10	2.37	2.61
*η*_0_ (Pa s)	4865	4580	5109	4481	5331

## Data Availability

Data is contained within the article.

## References

[1] Field G.J., Micic P., Bhattacharya S.N. (1999). Melt strength and film bubble instability of LLDPE/LDPE blends. Polym. Int..

[2] Ho K., Kale L., Montgomery S. (2002). Melt strength of linear low-density polyethylene/low-density polyethylene blends. J. Appl. Polym. Sci..

[3] Wagner M.H., Kheirandish S., Yamaguchi M. (2004). Quantitative analysis of melt elongational behavior of LLDPE/LDPE blends. Rheol. Acta.

[4] Stange J., Uhl C., Münstedt H. (2005). Rheological behavior of blends from a linear and a long-chain branched polypropylene. J. Rheol..

[5] Yamaguchi M., Shigehiko A. (1999). LLDPE/LDPE blends. I. Rheological, thermal, and mechanical properties. J. Appl. Polym. Sci..

[6] Delgadillo-Velázquez O., Hatzikiriakos S.G., Sentmanat M. (2008). Thermorheological properties of LLDPE/LDPE blends. Rheol. Acta.

[7] Micic P., Bhattacharya S.N., Field G. (1998). Transient elongational viscosity of LLDPE/LDPE blends and its relevance to bubble stability in the film blowing process. Polym. Eng. Sci..

[8] Dordinejad A.K., Jafari S.H. (2014). Miscibility analysis in LLDPE/LDPE blends via thermorheological analysis: Correlation with branching structure. Polym. Eng. Sci..

[9] Zhu H., Wang Y., Zhang X., Su Y., Dong X., Chen Q., Zhao Y., Geng C., Zhu S., Han C.C. (2007). Influence of molecular architecture and melt rheological characteristic on the optical properties of LDPE blown films. Polymer.

[10] Liu C., Wang J., He J. (2002). Rheological and thermal properties of m-LLDPE blends with m-HDPE and LDPE. Polymer.

[11] Ajji A., Sammut P., Huneault M.A. (2003). Elongational rheology of LLDPE/LDPE blends. J. Appl. Polym. Sci..

[12] McCallum T.J., Kontopoulou M., Park C.B., Muliawan E.B., Hatzikiriakos S.G. (2007). The rheological and physical properties of linear and branched polypropylene blends. Polym. Eng. Sci..

[13] Sugimoto M., Suzuki Y., Hyun K., Ahn K.H., Ushioda T., Nishioka A., Taniguchi T., Koyama K. (2006). Melt rheology of long-chain-branched polypropylenes. Rheol. Acta.

[14] Suleiman M.A. (2011). Rheological Investigation of the Influence of Short Chain Branching and Mw of LDPE on the Melt Miscibility of LDPE/PP Blends. Open Macromol. J..

[15] Dealy J.M., Larson R.G. (2006). Structure and Rheology of Molten Polymers.

[16] Nouri S., Dubois C., Lafleur P.G. (2015). Synthesis and characterization of polylactides with different branched architectures. J. Polym. Sci. Part B Polym. Phys..

[17] Nouri S., Dubois C., Lafleur P.G. (2015). Effect of chemical and physical branching on rheological behavior of polylactide. J. Rheol..

[18] Al-Itry R., Lamnawar K., Maazouz A. (2014). Reactive extrusion of PLA, PBAT with a multi-functional epoxide: Physico-chemical and rheological properties. Eur. Polym. J..

[19] Al-Itry R., Lamnawar K., Maazouz A. (2012). Improvement of thermal stability, rheological and mechanical properties of PLA, PBAT and their blends by reactive extrusion with functionalized epoxy. Polym. Degrad. Stab..

[20] Khoo H.H., Tan R.B.H. (2010). Environmental impacts of conventional plastic and bio-based carrier bags. Int. J. Life Cycle Assess..

[21] Pivsa-Art W., Pavasupree S., O-Charoen N., Insuan U., Jailak P., Pivsa-Art S. (2011). Preparation of Polymer Blends Between Poly (L-Lactic Acid), Poly (Butylene Succinate-Co-Adipate) and Poly (Butylene Adipate-Co-Terephthalate) for Blow Film Industrial Application. Energy Procedia..

[22] Fujimaki T. (1998). Processability and properties of aliphatic polyesters, “BIONOLLE”, synthesized by polycondensation reaction. Polym. Degrad. Stab..

[23] Xu J., Guo B. (2010). Poly (butylene succinate) and its copolymers: Research, development and dndustrialization. Biotechnol. J..

[24] Yoshikawa K., Ofuji N., Imaizumi M., Moteki Y., Fujimaki T. (1996). Molecular weight distribution and branched structure of biodegradable aliphatic polyesters determined by s.e.c.-MALLS. Polymer.

[25] Flory P.J., Fox T.G. (1951). Treatment of Intrinsic Viscosities. J. Am. Chem. Soc..

[26] Vega J., Aguilar M., Peón J., Pastor D., Martínez-Salazar J., Peon J., Pastor D., Martinez-Salazar J. (2002). Effect of long chain branching on linear-viscoelastic melt properties of polyolefins. e-Polymers.

[27] Garin M., Tighzert L., Vroman I., Marinkovic S., Estrine B. (2014). The influence of molar mass on rheological and dilute solution properties of poly(butylene succinate). J. Appl. Polym. Sci..

[28] McKee M.G., Unal S., Wilkes G.L., Long T.E. (2005). Branched polyesters: Recent advances in synthesis and performance. Prog. Polym. Sci..

[29] Zimm B.H., Stockmayer W.H. (1949). The Dimensions of Chain Molecules Containing Branches and Rings. J. Chem. Phys..

[30] Douglas J.F., Roovers J., Freed K.F. (1990). Characterization of Branching Architecture through “Universal” Ratios. Macromolecules.

[31] Wang G., Guo B., Li R. (2012). Synthesis, characterization, and properties of long-chain branched poly(butylene succinate). J. Appl. Polym. Sci..

[32] Kim E.U.N.K., Bae J.S., Im S.S., Kim B.C., Han Y.K. (2001). Preparation and Properties of Branched Polybutylenesuccinate. J. Appl. Polym. Sci..

[33] Wang L., Jing X., Cheng H., Hu X., Yang L., Huang Y. (2012). Blends of linear and long-chain branched poly(l -lactide)s with high melt strength and fast crystallization rate. Ind. Eng. Chem. Res..

[34] McLeish T.C.B. (2002). Tube theory of entangled polymer dynamics. Adv. Phys..

[35] Cox W.P., Merz E.H. (1958). Correlation of Dynamic and Steady Flow Viscosities. J. Polym. Sci..

[36] Carreau P.J. (1972). Rheological Equations from Molecular Network Theories. J. Rheol..

[37] Lehermeier H.J., Dorgan J.R. (2001). Melt rheology of poly(lactic acid): Consequences of blending chain architectures. Polym. Eng. Sci..

[38] Utracki L.A., Gendron R. (1984). Pressure Oscillation during Extrusion of Polyethylenes. II. J. Rheol..

[39] Yan D., Wang W.J., Zhu S. (1999). Effect of long chain branching on rheological properties of metallocene polyethylene. Polymer.

[40] Laun H.M. (1978). Description of the non-linear shear behaviour of a low density polyethylene melt by means of an experimentally determined strain dependent memory function. Rheol. Acta.

[41] Ramkumar D.H.S., Bhattacharya M. (1998). Steady shear and dynamic properties of biodegradable polyesters. Polym. Eng. Sci..

[42] Yilmazer U., Xanthos M., Bayram G., Tan V. (2000). Viscoelastic characteristics of chain extended/branched and linear polyethylene terephthalate resins. J. Appl. Polym. Sci..

[43] Trouton F.T. (1906). On the Coefficient of Viscous Traction and Its Relation to that of Viscosity. Proc. R. Soc. A Math. Phys. Eng. Sci..

[44] Hine P.J., Duckett A., Read D.J. (2007). Influence of Molecular Orientation and Melt Relaxation Processes on Glassy Stress-Strain Behavior in Polystyrene. Macromolecules.

[45] Liu J., Lou L., Yu W., Liao R., Li R., Zhou C. (2010). Long chain branching polylactide: Structures and properties. Polymer.

